# Minimally invasive percutaneous plate osteosynthesis versus intramedullary nail fixation for distal tibial fractures: a systematic review and meta-analysis

**DOI:** 10.1186/s13018-019-1479-0

**Published:** 2019-12-21

**Authors:** Bo Wang, Yang Zhao, Qian Wang, Bin Hu, Liang Sun, Cheng Ren, Zhong Li, Kun Zhang, Dingjun Hao, Teng Ma, Yao Lu

**Affiliations:** 1 Department of Orthopaedic Surgery, HongHui Hospital, Xi’an Jiaotong University, Xi’an, Shaan’xi Province China; 2 Department of Hematology, Xi’an GaoXin Hospital, Xi’an, Shaan’xi Province China

**Keywords:** Distal tibial fractures, Minimally invasive percutaneous plate osteosynthesis, Intramedullary nail, Meta-analysis

## Abstract

**Background:**

The treatment for distal tibial fractures remains controversial to date. Minimally invasive percutaneous plate osteosynthesis (MIPPO) and intramedullary nailing (IMN) are well-accepted and effective methods for distal tibial fractures, but these methods were associated with complications. This study aimed to assess and compare the clinical and functional outcomes in patients with distal tibial fractures treated with MIPPO or IMN.

**Methods:**

We systematically reviewed randomized controlled trials (RCTs) that compared MIPPO with IMN in patients with distal tibial fractures from inception till 15 August 2019. Also, quantitative summaries of time to reunion, rate of complications, and functional outcomes were evaluated.

**Results:**

The pooled results suggested that patients in the MIPPO group had a longer time to reunion with a mean difference of 1.21 weeks [*P* = 0.02; 95% confidence interval (CI) 0.16–2.26)] than those in the IMN group. The overall union complications and deep infection between IMN and MIPPO were similar (*P* > 0.05). IMN had a significantly low risk of wound complications [risk ratio (RR) = 0.51, *P* = 0.00, 95% CI 0.34–0.77)]. The pooled functional outcomes of the two groups remained controversial by different evaluating scores.

**Conclusions:**

Compared to MIPPO, IMN had a significantly low risk of wound complications and associated with limited time for reunion. Although the pooled functional outcomes of the two groups were controversial due to different evaluating scores, IMN was the preferred surgical technique than MIPPO for treating distal tibial fractures.

## Background

Tibial fractures are the most common bone fractures of the lower extremity, and high-energy injuries, such as motor vehicle trauma, falls, direct blow, and sports injury, frequently occur [1]. The distal tibial fractures constitute about 10–13% of all tibial fractures and are often associated with soft tissue injury [2]. These fractures can cause substantial disability in patients if no timely and proper treatment is provided.

Delayed union, nonunion, wound infection, and wound dehiscence are the most commonly observed complications due to the physiological characteristics of distal tibia, poor blood supply and decreased muscular cover anteriorly. Therefore, the ideal treatment for treating distal tibial fractures in patients remained controversial. Recently, minimally invasive percutaneous plate osteosynthesis (MIPPO) has been widely used owing to its technical advantages and satisfactory clinical outcomes [3, 4]. Similar to MIPPO, intramedullary nailing (IMN) also has been widely accepted as a treatment strategy for most of the open and closed tibial diaphyseal fractures [5]. However, both MIPPO and IMN were routinely associated with complications [6, 7]. A recent meta-analysis [8] based on five randomized controlled trials (RCTs) with 497 patients reported that MIPPO for distal tibial fractures is associated with a longer time to fracture union and an increased risk of wound complications. However, a stratified meta-analysis cannot be performed and a conclusion cannot be drawn on this research topic due to limited number of studies and smaller sample sizes.

Recently, several RCTs [9, 10] have focused on this topic in order to provide new evidence, allowing a systematic review and meta-analysis with more power of persuasion to be conducted. The present meta-analysis study aimed to explore and compare the outcomes of MIPPO and IMN for all types of distal tibial fractures.

## Methods

### Literature search

A systematic electronic search of databases such as PubMed, MEDLINE, Cochrane Library, and EMBASE was conducted to identify published studies from inception till 15 August 2019. Also, the bibliographies of all relevant studies and reviews identified were checked, and Google Scholar was searched for relevant studies. The process of selecting target articles was done according to the guidelines of Preferred Reporting Items for Systematic Reviews and Meta-Analyses (PRISMA) statement [11]. The individual and joint keywords such as “minimally invasive,” “intramedullary nail,” and “tibial fractures” were used for searching the literature.

### Eligibility criteria

The criteria for including studies into this meta-analysis were as follows: (1) RCTs that compared the outcomes of MIPPO and IM nail fixation in tibial fractures, (2) the study population were patients diagnosed with distal tibial fractures, (3) no evidence of polytrauma, (4) necessary data can be extracted or calculated from the original articles, and (5) articles published in English.

Case reports, letters, review articles, experimental nonrandomized studies, nonhuman studies, studies focusing on experiments in vitro, and studies not published in English were excluded from the analysis. If a study had duplicate publications, then the most recent publication was included. To minimize potential bias caused due to small sample sizes, studies with < 25 patients were excluded.

### Data extraction

All relevant articles from the abovementioned datasets were identified by two reviewers independently. For all the included studies, a customized and standardized form was used to extract the necessary information and a consensus was reached on all items by discussion with the abovementioned reviewers. For each included trial, the following details, such as authors, year of publication, study design, study population characteristics (e.g., age, sex, and nation), intervention/therapy characteristics (e.g., sample size for each group), and outcome assessment (e.g., type of outcome measure, length of follow-up and outcome measurements) were extracted.

### Bias risk assessment

The seven-category Cochrane Collaboration’s Risk of Bias tool [12] was used to assess the bias risk of the included trials. Trials were graded as unclear, high, or low risk of bias based on the following: (1) sequence generation, (2) allocation concealment, (3) blinding of personnel, (4) blinding of outcome assessor, (5) incomplete outcome data, (6) selective outcome reporting, and (7) other biases. For each included study, each item was judged as unclear if the author did not provide insufficient information to judge as low or high risk, or when there was no related information regarding the risk of bias item.

### Quality of studies assessment

Quality assessment of studies was performed independently and crosschecked by the two investigators mentioned above according to the Jadad scale, which independently assesses the methodological quality of each clinical trial [13]. For each study, a score of 0–5 was provided based on their performance of the three key methodological items: randomization, blinding, and accountability of all patients. One or two points were added for a “yes” to each of the randomization and blinding items, and one point was added for a “yes” to the item of accountability for all patients. A Jadad score of less than 3 points was used as the cut-off value for inclusion of a paper in this meta-analysis [14].

### Statistical analysis

Inverse variance method with random effects model was used to conduct pooled estimates of dichotomous outcomes, risk ratios (RRs), and 95% confidence intervals (CIs) of the included studies. The *I*^2^ statistic was used to assess the consistency of effect sizes to indicate the percentage of variability in the effect estimates because of true between-study variance rather than within-study variance. Heterogeneity was defined as low, moderate, and high with *I*^2^ values of 25%, 50%, and 75%, respectively [15]. To explore the sources of heterogeneity, all the enrolled studies were sequentially excluded to demonstrate the overall impact of individual studies, where *I*^2^ > 50%. Publication bias was assessed by the Begg rank correlation [16] and Egger weighted regression methods [17]. Stratified analyses were subsequently performed based on the characteristics of the study population and outcome. Review Manager (version5.3, The Cochrane Collaboration, Oxford, UK) was used for generating forest plots and statistical analyses. The Begg’s and Egger’s tests were assessed by STATA 15.0 (Stata Corporation, College Station, TX, USA). A *P* value of < 0.05 was considered to be statistically significant for all analyses.

## Results

### Study selection

**Fig. 1:**
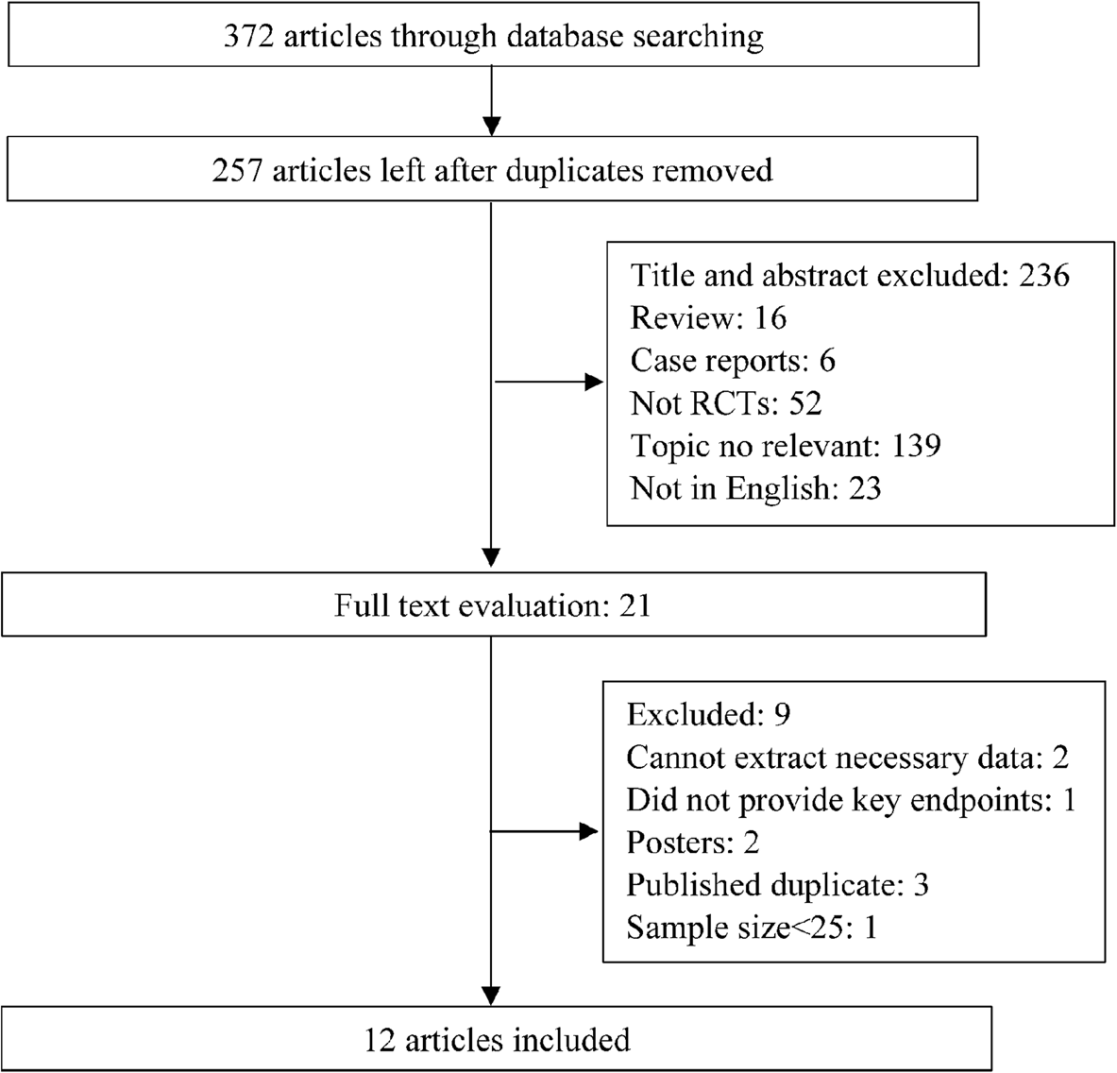
Flow chart of study selection process

The search strategy yielded 372 citations and 257 studies of these were excluded due to overlapping. Of the 372 studies, 236 studies were screened through titles or abstracts and were excluded as they did not meet the eligibility criteria. Finally, 12 RCTs [9, 10, 18–27] were considered eligible for data extraction and meta-analysis after reading the full-length manuscripts. A flow chart of the study selection process was shown in Fig. 1.

### Study characteristics

Thirteen RCTs with 900 patients were finally included in this meta-analysis and the characteristics of the included studies and patients were summarized in Additional file 1. The sample size of the included studies ranged from 25 to 321, and the studies were published between 2010 and 2018. Six studies were conducted in India [9, 10, 18–20], three in China [23, 26, 28], one in the UK [21], and one in Turkey [25] and the USA [24]. Nine studies [9, 10, 18–21, 25, 27] focused on closed or Gustilo patients and most of the studies [10, 19–21, 23–27] followed up participants for more than 12 months. Most of the studies reported the time to reunion of the tibia, except three studies [9, 10, 21].

### Risk of bias and quality assessment of studies

**Fig. 2:**
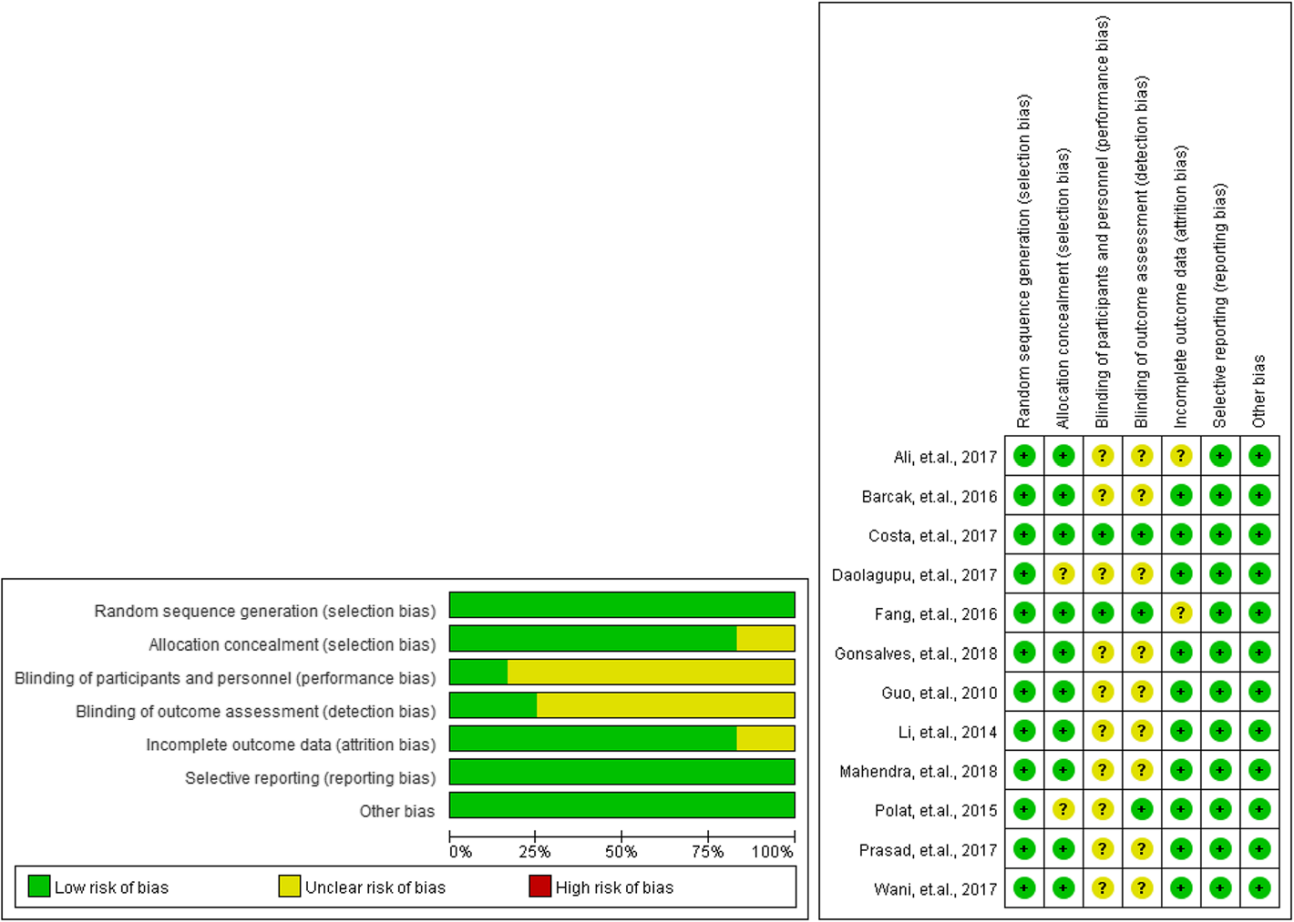
Risk of bias assessment of included studies

Most of the included studies exhibited a moderate risk of bias and an acceptable quality, and the overall risk of bias and quality were presented in Fig. 2 and Additional file 1. Two studies had full scores, and the remaining studies had a score of 3 as these studies did not undergo a blinding method by the Jadad scale. None of the included RCTs had a high risk by the seven-category Cochrane Collaboration’s Risk of Bias tool.

### Operation time

**Fig. 3:**
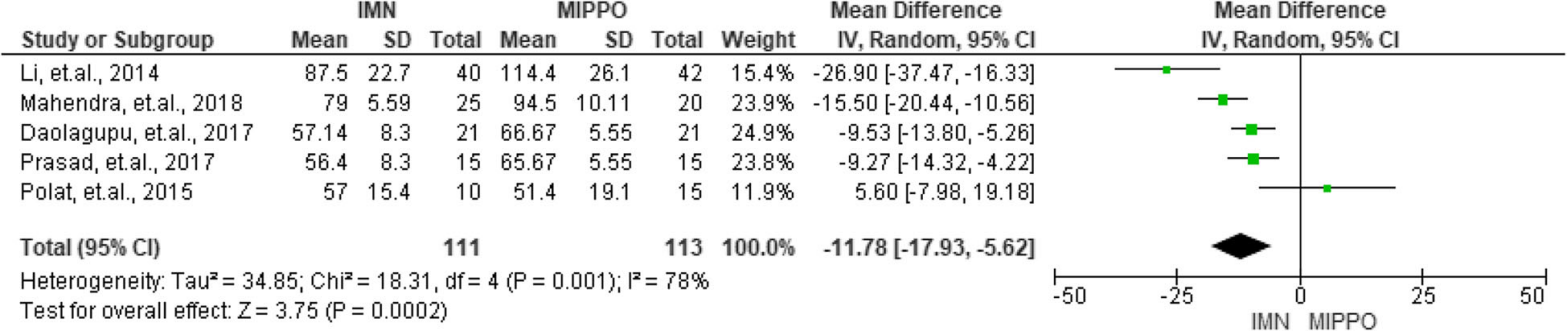
Summary of operation time of the included studies

As shown in Fig. 3, five studies reported and compared the detailed operation time of the IMN and MIPPO groups. The mean operation time in the IMN group ranged from 56.40 min to 87.50 min and that of MIPPO ranged from 51.40 min to 114.40 min. When the mean differences were pooled in these two groups, the summarized result demonstrated a moderate heterogeneity (*I*^2^ = 78%) and the MIPPO group demonstrated a significantly longer operation time with a mean difference of 11.78 min (*P* < 0.01).

### Time to union

**Fig. 4:**
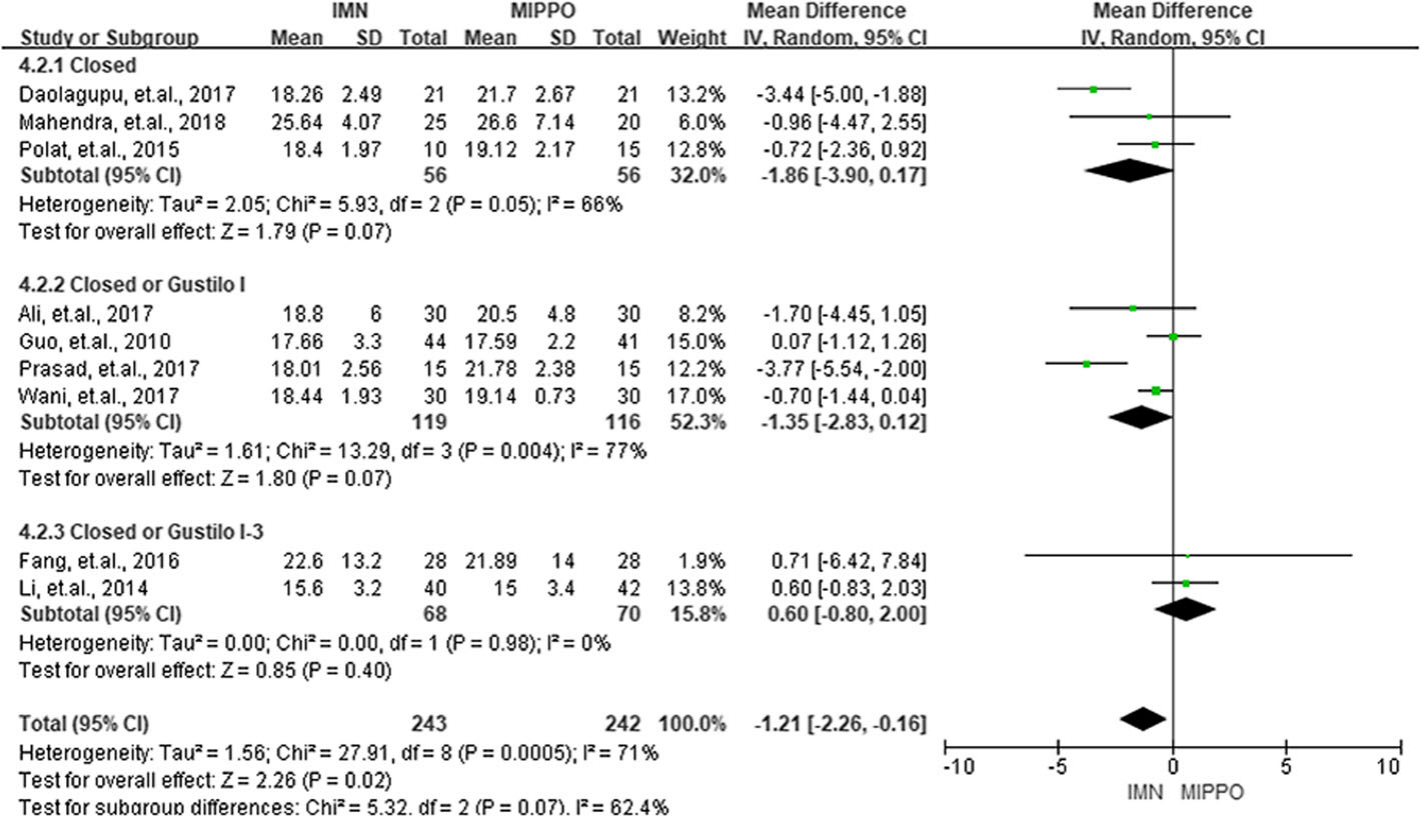
Summary of union time of the included studies

Of the12 RCTs, 9 studies provided data on union time and there are 243 patients in the IMN group and 242 patients in the MIPPO group. There was a moderate heterogeneity across the studies with *I*^2^ = 71%. The mean difference of IMN and MIPPO in each study ranged from − 3.77 weeks to 0.80 week, and the pooled results revealed that patients in MIPPO group required a longer reunion time than the IMN group with a mean difference of 1.21 weeks (*P* = 0.02, 95% CI 0.16–2.26). Subgroup analysis based on the type of fractures showed no statistically significant mean differences (*P* > 0.05) (Fig. 4).

### Complications

**Fig. 5:**
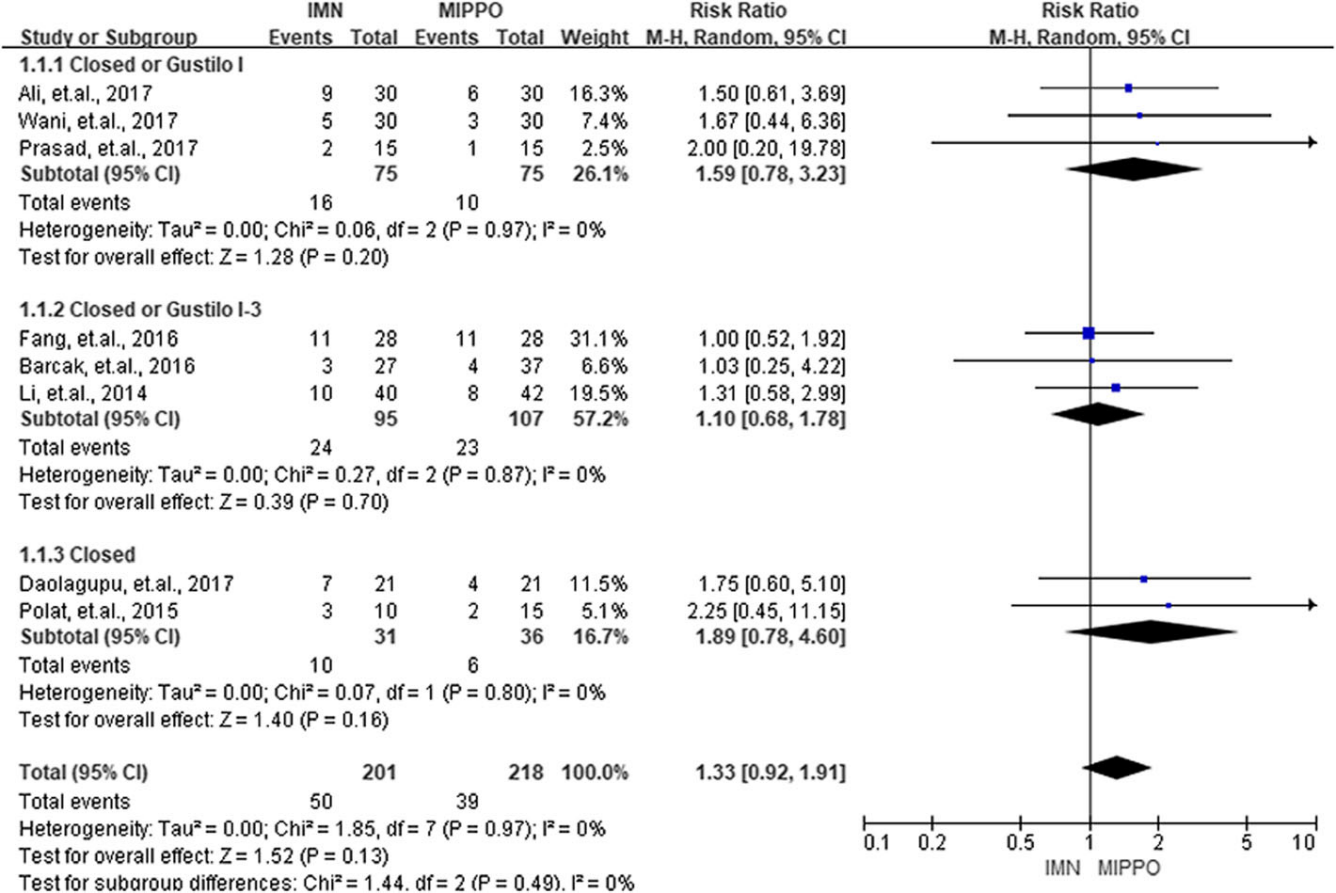
Summary of union complications of the included studies

**Fig. 6:**
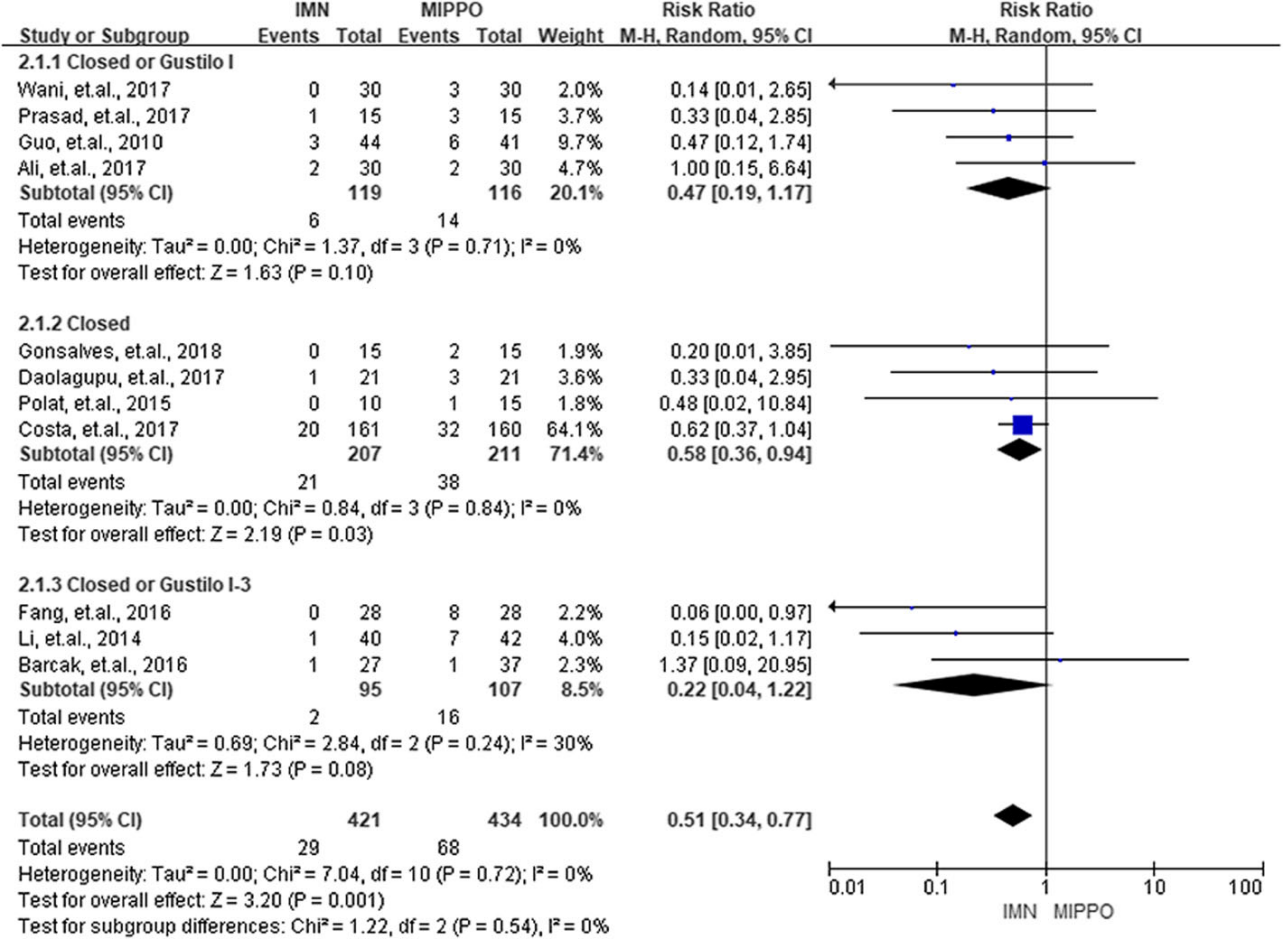
Summary of wound complications of the included studies

**Fig. 7:**
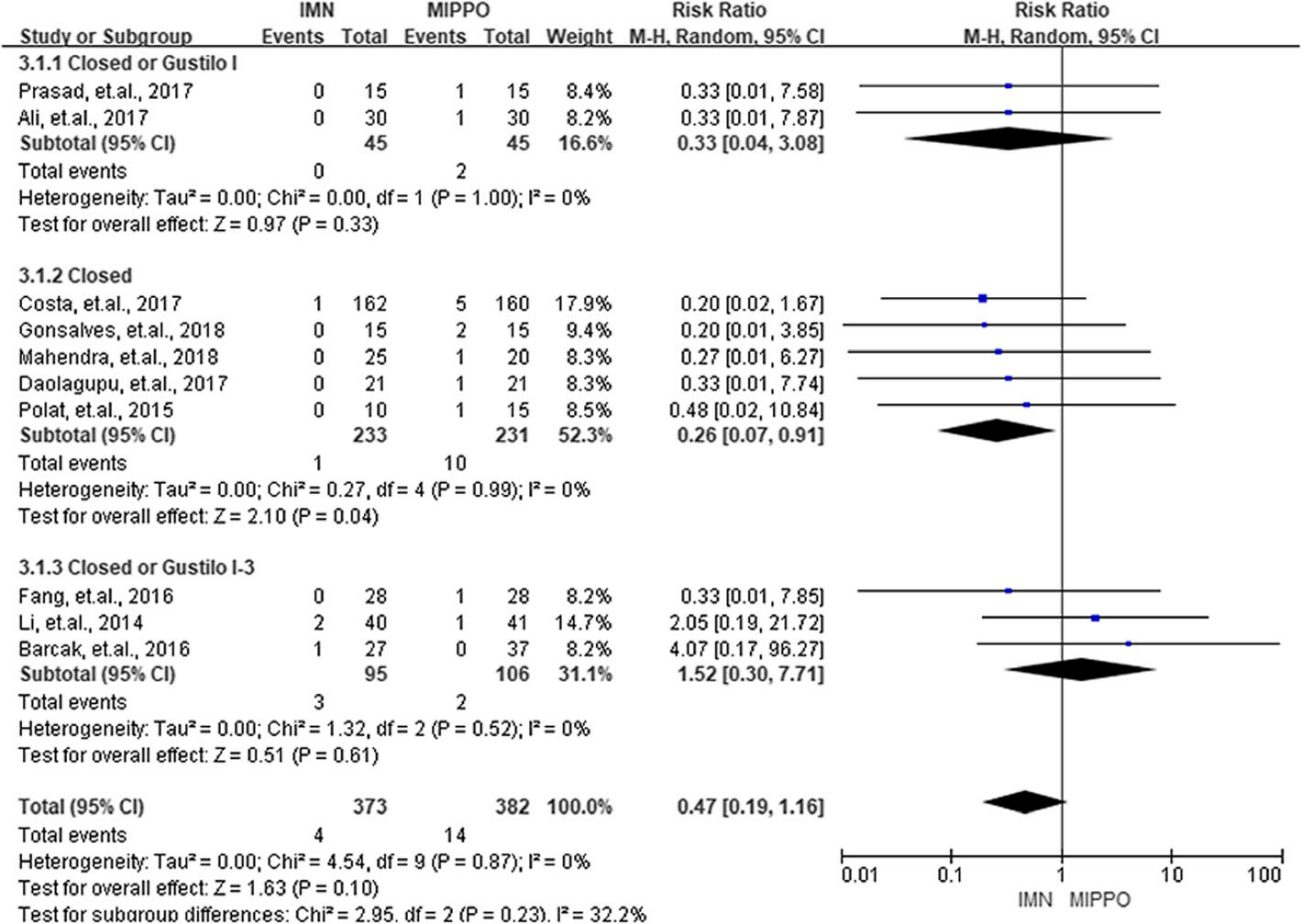
Summary of deep infections of the included studies

Complications in the current study were categorized into three groups, the union complications, wound complications, and deep infections. Union complications consisted of delayed union, non-union, and malunion. Eight studies with 419 patients reported union complications. The overall RR of union complications between IMN and MIPPO was 1.33 (*P* = 0.13, 95% CI 0.92–1.91), and showed no significant heterogeneity (*I*^2^ = 0%). Eleven studies with 856 patients and ten studies with 755 patients reported wound complications and deep infections, respectively. According to these results, IMN had a significantly lower risk of wound complications (RR = 0.51, *P* < 0.01, 95% CI 0.34–0.77) as well as deep infections (RR = 0.47, *P* = 0.10, 95% CI 0.19–1.16). No heterogeneity was observed during the process of summarizing wound complications (*I*^2^ = 0%) and deep infections (*I*^2^ = 0%). No statistical significance (*P* > 0.05) was observed when the patients were divided into three groups by the type of fractures for union complications, wound complications, and deep infections. The summarized results of union complications, wound complications, and deep infections were presented in Figs. 5, 6, and 7.

### Functional outcomes

**Fig. 8:**
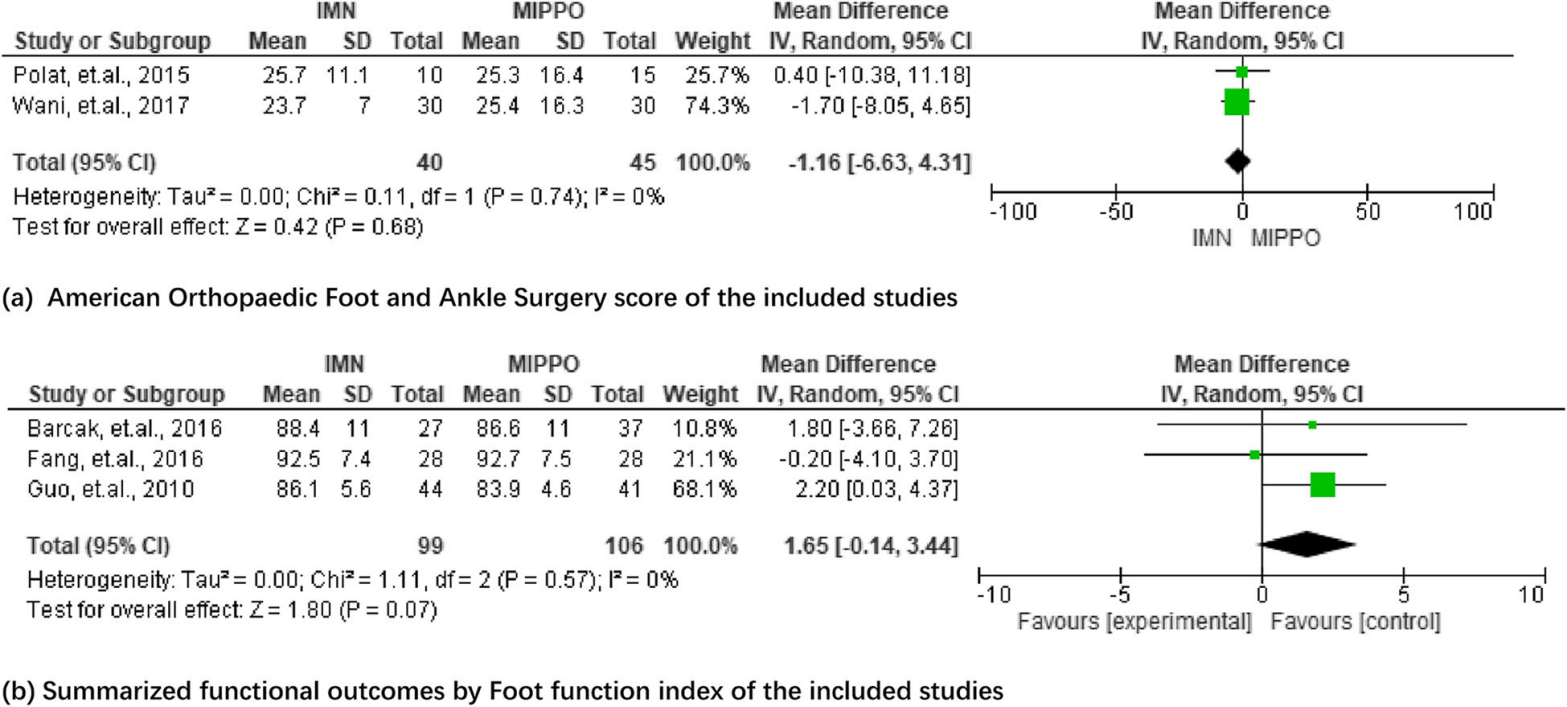
Summary of functional outcomes by various scores

Three articles provided functional outcomes based on the American Orthopaedic Foot and Ankle Surgery score (AOFAS) at 12 months after surgery. The foot function index (FFI) was employed to assess the functional outcomes in two studies. More data on functional outcomes of the included studies were presented in Additional file 1. The mean differences of summarized results for AOFAS and FFI at 12 months were 1.65 (*P* = 0.07, 95%CI − 0.14–3.44) and − 1.16 (*P* = 0.68, 95%CI − 6.63–4.31), respectively. The detailed data was presented in Fig. 8.

### Heterogeneity analyses

To explore the sources of heterogeneity, sensitivity analysis was performed by excluding each study sequentially based on the results of operation time and time to reunion. For pooled analysis on operation time, the results showed that after excluding the studies conducted by Li et al. [26] and Polat et al. [25], the heterogeneity was decreased to a slightly lower level (*I*^*2*^ = 51%, *P* = 0.13) (Additional file 1). For assessment on time to reunion, the *I*^*2*^ was decreased to 0% after excluding the studies conducted by Daolagupu et al. [19] and Prasad et al .[22] (Additional file 1).

### Publication bias

No potential publication bias among the included trials (*P* value of the analysis was more than 0.05) was observed according to Begg’s rank correlation analysis and Egger’s weighted regression analysis. The detailed potential publication bias of each analysis was shown in Additional file 1.

## Discussion

In the current meta-analysis of MIPPO versus IMN for distal tibial fractures, 13 RCTs with 924 patients were included. Compared with MIPPO, IMN was associated with less operation time and time to reunion. MIPPO significantly increased the rate of wound complications and the overall rate of union complications and deep infections were similar between the two groups. Similar functional outcomes were observed when evaluating the outcomes by AOFAS and FFI. The results also indicated that patients undergoing IMN might have a slightly better outcome according to DRI.

Recent meta-analyses studies [8] on this topic have drawn the conclusion that the MIPPO fixation technique is associated with a longer time to reunion and increased rate of wound complications. Although our study included more RCTs and larger sample size, the results remained similar. Both MIPPO and IMN are regarded as the two most commonly used methods for treating distal tibial fractures, but were associated with the development of complications [20, 28]. MIPPO is a noteworthy technique that manages fractures by avoiding some of the complications, including the union complications, wound complications, and deep infections, that are associated with conventional open plating methods [29, 30]. MIPPO aimed to preserve the osteogenic hematoma of the fracture and the nutritional arteries of the bone, while preventing the iatrogenic soft tissue from damage. The rate of wound complications and union time depended on several other factors apart from the surgical technique. Health condition of patients, skin and soft tissue contamination, operating room condition, and the timing of surgery all play an important role in the development of wound complications and increased union time. Previous studies also reported that wound complications normally delayed the wound healing process. A study conducted in Hong Kong, China [31], reported a late infection rate of 15% in patients undergoing MIPPO fixation for distal tibial fractures, and the implant was removed in 52% of patients due to skin impingement. Moreover, in the present study, MIPPO was associated with a longer operative time, due to complicated indirect reduction techniques of MIPPO.

The union complications including delayed union, nonunion, and malunion were similar in both IMN and MIPPO groups. A previous study also provided similar results [8]. The IMN spares the extraosseous blood supply, allows load sharing, and avoids extensive soft tissue dissection [32]. However, high attention must be paid on the technical difficulties with distal nail fixation during IMN and external fixation of distal tibial fractures might result in insufficient reduction, malunion, and pin tract infection.

As the studies included in this meta-analysis used more than seven different functional outcome scores to assess the functional outcomes, the pooled datasets included a smaller sample size. In the current study, the pooled scores of AOFAS and FFI remained controversial. AOFAS and FFI demonstrated similar outcomes in IMN and MIPPO groups. In contrast, the patients might have slightly better outcomes when using IMN according to DRI. Both pooled processes included limited studies and the dimension of each score was totally different, and so more RCTs should be conducted to evaluate studies with similar score criteria in the future.

For pooled results on operation time and time to union, a higher heterogeneity (*I*^*2*^ more than 70%) was observed. However, after excluding two studies, the heterogeneity was declined slightly and disappeared for the operation time and time to union, respectively. The possible explanations for this are as follows: firstly, as the remaining included studies were conducted in India, the heterogeneity on operation time might be caused due to the resource of subjects; and secondly, the sample size of the excluded two studies was 15 and 21 participants in each group. Therefore, the sample size of the study could also be associated with heterogeneity.

It is noteworthy to consider the limitations of the present meta-analysis when interpreting the results. Firstly, most of the included studies had limited sample sizes, and so more subgroups or sensitivity analyses could not be conducted. Secondly, most of the studies did not match the participants by age or sex. Therefore, the mean age and the sex ratio of each RCT varied largely, causing heterogeneity and reducing the stability of the results. Moreover, the RCTs included fracture patients with varied severities and the fracture pattern of each included study was significantly complex, causing heterogeneity of the results. Thirdly, the process of evaluating functional outcomes is relatively promiscuous. More than seven scores including DRI score, OMAS, AOFAS score, FFI, and Johner and Wruh’s criteria were employed to assess the functional outcomes. Therefore, only few studies can be included for combining the functional outcomes. Finally, potential language bias might exist as our literature search considered only articles published in English.

## Conclusions

In conclusion, our meta-analysis compared IMN versus MIPPO for the treatment of tibial fractures. The results demonstrated high rate of wound complications, longer operation time, and a longer time to union with MIPPO when compared to IMN. Regarding the functional outcomes, IMN and MIPPO demonstrated similar findings using AOFAS and FFI. Based on DRI, patients might have slightly better outcomes when using IMN. In total, IMN demonstrated had more advantages than MIPPO and was preferred for patients with distal tibial fractures. In future, larger RCTs and RCTs by matching age, sex, and severity degrees of the patients should be conducted for detecting important differences.

## Supplementary information

**Additional file 1: Table S1.** Study participants’ characteristics of the included studies. **Table S2.** Jadad score for included studies. **Table S3.** The functional outcomes of the included studies. **Table S4.** Publication bias of summarized outcomes. **Figure S1.** Pooled analysis of operation time after excluding two studies. **Figure S2.** Pooled analysis of time to reunion after excluding two studies.

## Data Availability

Data sharing is not applicable to this article as no datasets were generated or analyzed during the current study.

## Abbreviations

IMN: Intramedullary nailing
MIPPO: Minimally invasive percutaneous plate osteosynthesis
RCTs: Randomized controlled trial
