# Supplementary material for: Minimally invasive percutaneous plate osteosynthesis versus intramedullary nail fixation for distal tibial fractures: a systematic review and meta-analysis
Source: J Orthop Surg Res. 2019 Dec 21;14:456. doi: 10.1186/s13018-019-1479-0 (PMC6925456; doi:10.1186/s13018-019-1479-0)
Supplement: Supplementary file 1 — Additional file 1: Table S1. Study participants’ characteristics of the included studies. Table S2. Jadad score for included studies. Table S3. The functional outcomes of the included studies. Table S4. Publication bias of summarized outcomes. Figure S1. Pooled analysis of operation time after excluding two studies. Figure S2. Pooled analysis of time to reunion after excluding two studies. [file 13018_2019_1479_MOESM1_ESM.docx]

**Table S1. Study participants’ characteristics of the included studies**

| **Study included** | **Country** | **Gender (M/F)** | | **Age, years (means±SD)** | | **Fracture type** | **AO/OTA classification** | |
| --- | --- | --- | --- | --- | --- | --- | --- | --- |
|  |  | **IMN** | **MIPPO** | **IMN** | **MIPPO** |  | **IMN** | **MIPPO** |
| Guo, et.al., 2010 | China | 26/18 | 24/17 | 44.20 (27.00-70.00)^a^ | 44.40 (23.00-69.00)^a^ | Closed or Gustilo I | 43-A1: 13  43-A2: 16  43-A3: 15 | 43-A1: 13  43-A2: 12  43-A3: 16 |
| Li, et.al., 2014 | China | 41/5 | 38/8 | 44.00 | 43.00 | Closed, Gustilo I, or II | 42-A: 33  42-B: 8  42-C: 5 | 42-A: 37  42-B: 7  42-C: 2 |
| Polat, et.al., 2015 | Turkey | 7/3 | 9/6 | 34.00±9.70 | 36.40±10.70 | Closed | 42-A1: 6  42-A2: 3  42-A3: 1 | 42-A1: 11  42-A2: 1  42-A3: 3 |
| Fang, et.al., 2016 | China | 19/9 | 21/7 | 35.00±9.20 | 38.60±7.50 | Closed, Gustilo I, or II | 42-A: 16  42-B: 8  42-C: 4 | 42-A: 15  42-B: 10  42-C: 3 |
| Barcak, et.al., 2016 | United States | 18/9 | 25/12 | 46.00 (26.00-66.00) ^a^ | 48.50 (18.00-82.00) ^a^ | Closed or Gustilo I-3 | 43-A1: 6  43-A2: 5  43-A3: 3  43-C1: 4  43-C2: 9 | 43-A1: 0  43-A2: 20  43-A3: 6  43-C1: 7  43-C2: 4 |
| Ali, et.al., 2017 | India | 23/7 | 21/9 | 40.40±12.30 | 41.90±13.50 | Closed or Gustilo I | 42-A: 17  42-B: 6  42-C: 0  43-A: 8 | 42-A: 16  42-B: 5  42-C: 1  43-A: 8 |
| Prasad, et.al., 2017 | India | 12/3 | 13/2 | 35.67+8.12 | 36.57±7.68 | Closed or Gustilo I | 43-A1: 8  43-A2: 5  43-A3: 2 | 43-A1: 9  43-A2: 4  43-A3: 2 |
| Wani, et.al., 2017 | India | 22/8 | 20/10 | 36.40±9.70 | 38.40±8.70 | Closed or Gustilo I | 42-A1: 18  42-A2: 9  42-A3: 3 | 42-A1: 20  42-A2: 2  42-A3: 8 |
| Costa, et.al., 2017 | United Kingdom | 96/65 | 101/59 | 44.30±16.30 | 45.80±16.30 | Closed | NA | NA |
| Daolagupu, et.al., 2017 | India | 17/4 | 15/6 | 35.19±9.22 | 39.09±10.13 | Closed | 43-A1: 11  43-A2: 6  43-A3: 4 | 43-A1: 10  43-A2: 9  43-A3: 2 |
| Gonsalves, et.al., 2018 | India | 13/2 | 14/1 | 40.00 | 43.00 | Closed | 43-A0: 15 | 43-A0: 15 |
| Mahendra, et.al., 2018 | India | 16/9 | 12/8 | 41.04±14.07 | 41.90±15.27 | Closed | NA | NA |

Abbreviations: M/F, male/female; SD, standard deviation; IMN, intramedullary nailing; MIPPO, minimally invasive percutaneous plate osteosynthesis; NA, not available.

**Table S2. Jadad score for included studies**

| **Study included** | **Randomization ^a^** | **Blinding ^b^** | **An account of all patients ^c^** | **Overall** |
| --- | --- | --- | --- | --- |
| Guo, et.al., 2010 | 2 | 0 | 1 | 3 |
| Mauffrey, et.al., 2012 | 2 | 2 | 1 | 5 |
| Li, et.al., 2014 | 2 | 0 | 1 | 3 |
| Polat, et.al., 2015 | 2 | 0 | 1 | 3 |
| Fang, et.al., 2016 | 2 | 2 | 1 | 5 |
| Barcak, et.al., 2016 | 2 | 0 | 1 | 3 |
| Ali, et.al., 2017 | 2 | 0 | 1 | 3 |
| Prasad, et.al., 2017 | 2 | 0 | 1 | 3 |
| Wani, et.al., 2017 | 2 | 0 | 1 | 3 |
| Costa, et.al., 2017 | 2 | 2 | 1 | 5 |
| Daolagupu, et.al., 2017 | 2 | 0 | 1 | 3 |
| Gonsalves, et.al., 2018 | 2 | 0 | 1 | 3 |
| Mahendra, et.al., 2018 | 2 | 0 | 1 | 3 |

^a^, 1 point if randomization is mentioned, 1 additional point if the method of randomization is appropriate. Deduct 1 point if the method of randomization is inappropriate (minimum 0).

^b^, 1 point if blinding is mentioned, 1 additional point if the method of blinding is appropriate. Deduct 1 point if the method of blinding is inappropriate (minimum 0).

^c^, the fate of all patients in the trial is known. If there are no data, the reason is stated.

**Table S3. The functional outcomes of the included studies**

| **Study included** | **Functional outcomes** | | | |
| --- | --- | --- | --- | --- |
|  | **Time (months)** | **Evaluation criterion** | **IMN** | **MIPPO** |
| Guo, et.al., 2010 | 12 | AOFAS | 86.10(83.70- 88.60) ^a^ | 83.90(81.70-86.10) ^a^ |
| Li, et.al., 2014 | 12 | Mazur ankle score | 0.87 (35/40) | 0.90 (38/42) |
| Polat, et.al., 2015 | 12 | FFI | 25.70±11.10 | 25.30±16.40 |
| Fang, et.al., 2016 | 28 | AOFAS | 92.50±7.40 | 92.70±7.50 |
| Barcak, et.al., 2016 | 12 | AOFAS | 88.40±11.00 | 86.60±11.00 |
| Ali, et.al., 2017 | 9 | Teeny and Wiss clinical assessment criteria | 86.90±10.00 | 88.60±6.60 |
| Prasad, et.al., 2017 | NA | NA | NA | NA |
| Wani, et.al., 2017 | 12 | FFI | 23.70±7.00 | 25.40±16.30 |
| Costa, et.al., 2017 | 3 | DRI | 44.20(40.80-47.60) ^a^ | 52.60(49.30-55.90) ^a^ |
|  | 12 | DRI | 23.10(18.90-27.20) ^a^ | 24.00(19.70-28.30) ^a^ |
| Daolagupu, et.al., 2017 | 12 | Johner and Wruh’s criteria: good | 14.28% | 28.57% |
|  | 12 | Johner and Wruh’s criteria: excellent | 57.14% | 52.38% |
| Gonsalves, et.al., 2018 | 6-22 | Johner and Wruh’s criteria: good | 13.33% | 20.00% |
|  | 6-22 | Johner and Wruh’s criteria: excellent | 53.33% | 40.00% |
| Mahendra, et.al., 2018 | 24 | OMS | 82.40±11.50 | 81.50±12.90 |

Abbreviations: IMN, intramedullary nailing; MIPPO, minimally invasive percutaneous plate osteosynthesis; NA, not available; AOFAS, American Orthopaedic Foot and Ankle Surgery score; DRI, Disability Rating Index score; OMS, Olerud Molander score; FFI, Foot function index.

^a^, 95% confidence interval.

**Table S4. Publication bias of summarized outcomes**

| **Outcomes** | **Begg (*P* value)** | **Egger (*P* value)** |
| --- | --- | --- |
| Summarized time to union. | 0.50 | 0.89 |
| Summarized union complications | 0.62 | 0.55 |
| Summarized wound complications | 0.78 | 0.65 |
| Summarized deep infections | 0.91 | 0.87 |
| Summarized operation time | 0.50 | 0.38 |
| Summarized functional outcome by AOFAS | 0.87 | 0.65 |
| Summarized functional outcome by FFI | 0.76 | 0.54 |

Abbreviations: AOFAS, American Orthopaedic Foot and Ankle Surgery score; DRI, Disability Rating Index score; FFI, Foot function index.

_
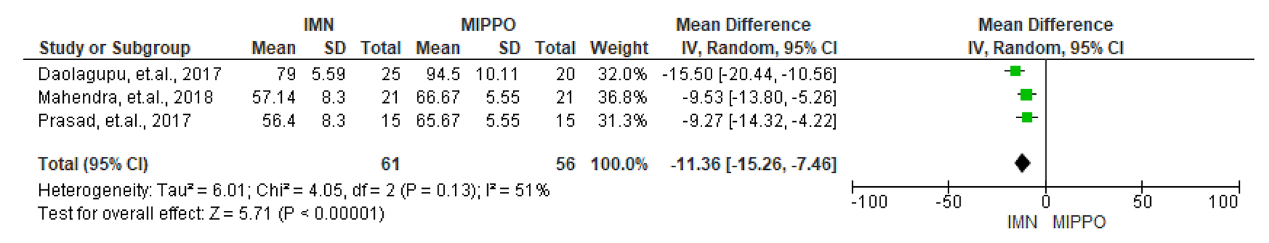
_

Figure S1 Pooled analysis of operation time after excluding two studies.


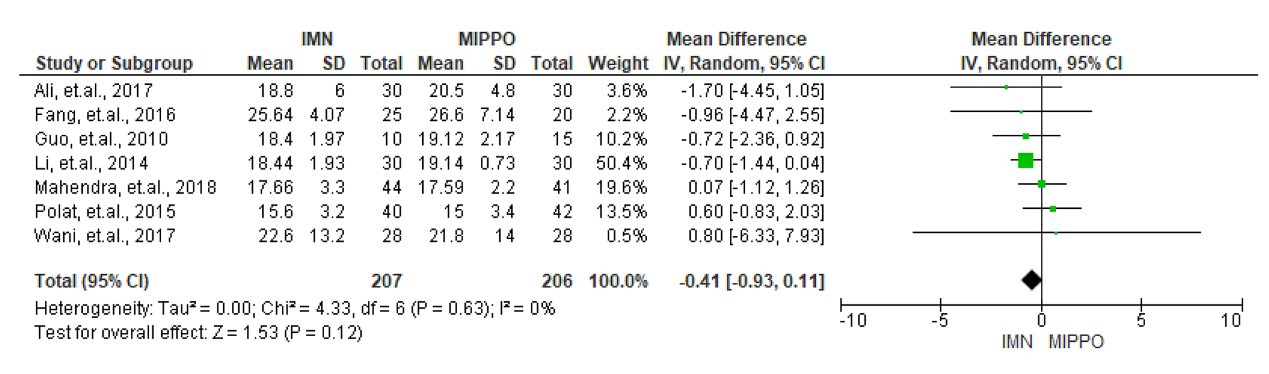


Figure S2 Pooled analysis of time to reunion after excluding two studies.
